# Dynamic Measurement of Hemodynamic Parameters and Cardiac Preload in Adults with Dengue: A Prospective Observational Study

**DOI:** 10.1371/journal.pone.0156135

**Published:** 2016-05-19

**Authors:** Vipa Thanachartwet, Anan Wattanathum, Duangjai Sahassananda, Petch Wacharasint, Supat Chamnanchanunt, Ei Khine Kyaw, Akanitt Jittmittraphap, Mali Naksomphun, Manoon Surabotsophon, Varunee Desakorn

**Affiliations:** 1 Department of Clinical Tropical Medicine, Faculty of Tropical Medicine, Mahidol University, Bangkok 10400, Thailand; 2 Pulmonary and Critical Care Division, Department of Medicine, Phramongkutklao Hospital, Bangkok 10400, Thailand; 3 Information Technology Unit, Faculty of Tropical Medicine, Mahidol University, Bangkok 10400, Thailand; 4 Critical Care Division, Department of Anesthesiology, Phramongkutklao Hospital, Bangkok 10400, Thailand; 5 Department of Microbiology and Immunology, Faculty of Tropical Medicine, Mahidol University, Bangkok 10400, Thailand; 6 Hospital for Tropical Diseases, Faculty of Tropical Medicine, Mahidol University, Bangkok 10400, Thailand; 7 Pulmonary and Critical Care Division, Department of Medicine, Ramkhamhaeng Hospital, Bangkok 10240, Thailand; Institute of Tropical Medicine (NEKKEN), Nagasaki University, JAPAN

## Abstract

Few previous studies have monitored hemodynamic parameters to determine the physiological process of dengue or examined inferior vena cava (IVC) parameters to assess cardiac preload during the clinical phase of dengue. From January 2013 to July 2015, we prospectively studied 162 hospitalized adults with confirmed dengue viral infection using non-invasive cardiac output monitoring and bedside ultrasonography to determine changes in hemodynamic and IVC parameters and identify the types of circulatory shock that occur in patients with dengue. Of 162 patients with dengue, 17 (10.5%) experienced dengue shock and 145 (89.5%) did not. In patients with shock, the mean arterial pressure was significantly lower on day 6 after fever onset (*P* = 0.045) and the pulse pressure was significantly lower between days 4 and 7 (*P<*0.05). The stroke volume index and cardiac index were significantly decreased between days 4 and 15 and between days 5 and 8 after fever onset (*P*<0.05), respectively. A significant proportion of patients with dengue shock had an IVC diameter <1.5 cm and IVC collapsibility index >50% between days 4 and 5 (*P*<0.05). Hypovolemic shock was observed in 9 (52.9%) patients and cardiogenic shock in 8 (47.1%), with a median (interquartile range) time to shock onset of 6.0 (5.0–6.5) days after fever onset, which was the median day of defervescence. Intravascular hypovolemia occurred before defervescence, whereas myocardial dysfunction occurred on the day of defervescence until 2 weeks after fever onset. Hypovolemic shock and cardiogenic shock each occurred in approximately half of the patients with dengue shock. Therefore, dynamic measures to estimate changes in hemodynamic parameters and preload should be monitored to ensure adequate fluid therapy among patients with dengue, particularly patients with dengue shock.

## Introduction

Dengue is a mosquito-borne viral disease in humans caused by one of four dengue virus (DENV) serotypes [1]. Dengue is rapidly becoming widespread across the globe [1]. Approximately 500,000 people with severe dengue require hospitalization, and 2.5% of those affected die annually [2]. Recently, there was an epidemic shift in the age pattern of dengue, from children to adults, accompanied by an increase in severity [3–5]. The common complications of dengue in adults are bleeding and organ impairment [6], but severe plasma leakage leading to circulatory shock is a common cause of death [7]. Circulatory shock in dengue occasionally occurs during the critical phase or defervescence period [1, 3]. Previous reports showed cardiac involvement in adults with dengue, including myocardial dysfunction (42–47%), arrhythmias (29–63%), and myocarditis (15–29%) [8–11].

Beginning in 2009, the revised World Health Organization (WHO) treatment guidelines were used for the diagnosis and management of dengue [3]. However, the parameters recommended for the diagnosis and management of dengue shock could not differentiate between the types of circulatory failure, i.e., hypovolemic versus cardiogenic shock [1, 3]. At present, prompt recognition and early identification of the type of circulatory shock and appropriate resuscitation using fluid therapy and/or vasopressors are the cornerstones of intensive care medicine [12, 13]. Hemodynamic or physiologic monitoring plays an important role in diagnosing the type of circulatory shock present based on the pathophysiologic process, enabling proper patient management [13].

The primary hemodynamic parameters include heart rate (HR) and blood pressure (BP), while the advanced hemodynamic parameters include stroke volume (SV), cardiac output (CO), and total peripheral resistance (TPR) [14]. The measurement technique for hemodynamic parameters, particularly CO, previously utilized an invasive pulmonary artery catheter and an arterial or central venous catheter for gravely ill patients [14, 15]. Recently, non-invasive methods for the evaluation of hemodynamic parameters have been developed, and the results of these techniques are highly correlated with those of invasive methods for determining the type of circulatory shock [14]. Similarly, a number of bedside ultrasonography protocols to estimate the cardiac preload have become widely used for determining the type of circulatory shock [16–17]. In dengue, the dynamic measurement of hemodynamic parameters, for determining the pathophysiologic process, and inferior vena cava (IVC) parameters, to estimate cardiac preload during the clinical phase of dengue, has not been extensively performed in adults. We hypothesized that hypovolemic and/or cardiogenic shock might be causes of dengue shock and that hemodynamic and IVC parameters differ between patients with and without shock along a specific time course.

## Objectives

The aims of this study were (1) to identify changes in hemodynamic and IVC parameters and (2) to identify the types of circulatory shock present among patients with dengue using non-invasive bedside techniques.

## Methods

### Ethical statement

This study was approved by the Ethics Committee of the Faculty of Tropical Medicine, Mahidol University, Bangkok, Thailand. The procedure described by the Standards for the Reporting of Observation Studies in Epidemiology (STROBE) was followed [18]. Written informed consent was obtained from all patients and from patients’ guardians in the case of patients under 18 years of age. Data were made anonymous before analysis.

### Study design and population

This prospective observational study was conducted at the Hospital for Tropical Diseases, Faculty of Tropical Medicine, Mahidol University in Bangkok, Thailand. Patients admitted to the hospital between January 2013 and July 2015 who met the study criteria were approached for participation. The study’s inclusion criteria were: (1) patients at least 15 years old; (2) presenting with clinical criteria for dengue, defined as acute fever with at least two of the following symptoms: headache, ocular pain, myalgia, arthralgia, rash, a positive tourniquet test (defined as the presence of ≥20 petechiae per 1 square inch), or leukopenia (defined as a white blood cell count <5.0 × 10^3^ cells/μL); and (3) having a confirmed dengue viral infection, defined as positive tests of either (a) viral nucleic acid using reverse-transcriptase polymerase chain reaction (RT-PCR) from serum samples on admission, (b) micro-neutralization test or (c) specific dengue IgM and IgG antibodies using enzyme-linked immunosorbent assays (ELISA) from serum samples on admission and at least two weeks later.

Upon admission, dengue viral RNA was detected from the patients’ sera using two rounds of PCR, as described by Lanciotti *et al*. [19] with modifications by Reynes *et al*. [20]. Samples were detected using a PureLink® Viral RNA/DNA Mini Kit (Invitrogen™, USA) according to the manufacturer’s instructions. Serum samples at admission and at two weeks after fever onset were assayed for serotype-specific DENV using the micro-neutralization test described by Vorndam *et al*. [21], with slight modification by Putnak *et al*. [22]; IgM and IgG antibodies for DENV were detected from these samples also, using capture ELISA as described by Innis *et al*. [23].

Patients with a history of underlying medical illness, including diabetes mellitus, hypertension, hyperlipidemia, cardiovascular diseases, cerebrovascular diseases, epilepsy, lung diseases, liver diseases, kidney diseases, autoimmune diseases, and human immunodeficiency virus (HIV) infection and malignancy, mixed infection, or who were currently pregnant were excluded. Hemodynamic and IVC parameters were recorded at admission and every 24 hours during hospitalization. Laboratory tests, including complete blood count and blood chemistry, were performed at admission. To exclude other infections, two microbiology blood cultures, urinalysis, and posterior-anterior chest radiography were routinely performed in hospitalized patients at admission. Other diagnostic tests for infectious diseases were performed when clinical findings were suspicious. Cardiac biomarkers, including troponin T and N-terminal pro-brain natriuretic peptide (NT-proBNP), electrocardiography (ECG), and posterior-anterior chest radiographs were evaluated when body temperature <37.8°C for 24–48 hours. The type of fluid therapy, amount of fluid intake, and urine output were recorded every 24 hours during hospitalization. All dengue patients in this study received standard care according to the WHO treatment guidelines for dengue by the treating physicians [1, 3]. The severity of dengue was summarized on discharge.

At the 2-week follow-up, complete blood count, serum creatinine, and hemodynamic and IVC data were recorded and used as a baseline for each patient. Patient data, including baseline characteristics, clinical parameters, laboratory findings, hemodynamic parameters, IVC parameters, cardiac biomarkers, ECG, chest radiography, type of fluid, daily fluid intake, and urine output per day were recorded on a pre-defined case-report form.

### Hemodynamic assessment

Hemodynamic parameters, including the primary hemodynamic parameters HR, mean arterial pressure (MAP), and BP, as well as the advanced hemodynamic parameters cardiac index (CI), total peripheral resistance index (TPRI), and stroke volume index (SVI), were recorded using a non-invasive CO monitor (NICOM^TM^, Cheetah Medical Ltd, Portland, USA). This monitor was shown to be non-invasive with acceptable accuracy and precision (r = 0.78–0.82) in previous studies [24–28]. These hemodynamic parameters were recorded from an average of 3 readings taken at intervals of 1–3 minutes.

### Central venous pressure assessment

Evaluation of the IVC using two dimensional (2D) bedside ultrasonography (GE Healthcare^TM^, General Electric Company, UK) was performed by a well-trained investigator for assessing central venous pressure (CVP). The IVC parameters, including the IVC diameter (IVCd) and collapsibility index (IVCc), have been previously collected in spontaneously breathing patients for use in determining the CVP level, to help differentiate between types of circulatory shock and guide fluid therapy [29–31]. A cardiac transducer (1.5–3.5 MHz, GE Logiq S6, UK) was used for all examinations of IVC images. The IVC was imaged in a long axis view with the cardiac transducer in the subxiphoid window. The intrahepatic segment of the IVC was visualized as it entered the right atrium. The M-mode cursor was used to create a time-motion image of the IVCd. The IVCd data were collected during 20 seconds of spontaneous breathing, approximately 2–3 cm from the junction of the IVC and right atrium, using the anterior-posterior dimension. The maximum IVCd (IVCdmax) was measured at the end-expiratory phase and the minimum IVCd (IVCdmin) was measured at the end-inspiratory phase over a single respiratory cycle. The IVCc was calculated as the difference between IVCdmax and IVCdmin divided by the IVCdmax, expressed as a percentage ([IVCdmax-IVCdmin]/IVCdmax × 100) [29]. A previous meta-analysis found that the mean IVCd in hypotensive groups ranged from 0.56–1.55 cm and the mean IVCd in normotensive groups ranged from 1.07–2.90 cm [30]. The IVCd was significantly lower in the hypovolemic groups than in the normotensive groups with a mean difference (95% confidence interval, 95% CI) of 0.63 (0.60–0.65) cm [30]. At the 2-week follow-up, our patients had a median (95% CI) IVCd of 1.6 (1.5–1.7) cm. At admission, the IVCd was significantly lower than that at the 2-week follow-up (*P* = 0.002) with a median difference (95% CI) of 0.1 (0–0.2) cm. Thus, we used the lower limit of 95% CI (1.5 cm) of the median IVCd as a cutoff value, and IVCd ˂1.5 cm was defined as a narrow IVCd in our study. Other previous studies also showed that IVCd ˂1.5 cm with IVCc >50% correspond to a CVP ≤5 mmHg [31, 32].

### Modified approach protocol for determining the type of circulatory shock

A modified approach protocol was performed among patients with dengue shock to determine the type of circulatory shock that occurred. In our study, a combination of non-invasive CO monitoring and 2D bedside ultrasonography was used for determining the type of circulatory shock that occurred, because this method is non-invasive, readily available, easy to use, and has acceptable device accuracy; furthermore, this method can potentially guide fluid therapy. The hemodynamic parameters, including CI and TPRI, were used to determine the type of circulatory shock present among patients with circulatory failure, as described previously [33]. In addition, IVCd and IVCc were also used to differentiate between the types of circulatory shock, according to the Abdominal and Cardiac Evaluation with Sonography in Shock (ACES) criteria [16]. Hypovolemic shock was defined as CI <2.5 L/min/m^2^ and TPRI >2400 dynes∙sec/cm^5^/m^2^ with a narrow IVCd (<1.5 cm) and IVCc >50%. Cardiogenic shock was defined as CI <2.5 L/min/m^2^ and TPRI >2400 dynes∙sec/cm^5^/m^2^ with a normal IVCd (≥1.5 cm) and IVCc ≤50%. Septic shock was defined as CI >4.0 L/min/m^2^ and TPRI <1900 dynes∙sec/cm^5^/m^2^ with narrow IVCd (<1.5 cm) and IVCc >50%.

Dengue shock was defined as plasma leakage with shock. Plasma leakage was defined as a ≥20% increase in hematocrit (Hct) above baseline, with the baseline defined as the Hct level during the 2-week follow-up period, or clinical fluid accumulation manifested by pleural effusion, ascites, or serum albumin <3.5 g/dL. Shock was defined as (1) a rapidly weak pulse with a pulse pressure (PP) <20 mmHg [1, 3] or (2) a systolic BP of <90 mmHg with evidence of tissue hypoperfusion, i.e. <0.5 mL/kg/h decrease in urine output, impaired consciousness, aspartate aminotransferase (AST) >1,000 IU/L, alanine aminotransferase (ALT) >1,000 IU/L, or cold or clammy skin [1, 3, 34]. Recurrent shock was said to occur when shock patients had a systolic BP of <90 mmHg with evidence of tissue hypoperfusion after receiving adequate fluid therapy according to the WHO treatment guidelines for dengue [1, 3]. The adequacy of fluid therapy was evaluated by estimating the jugular venous pressure and performing the orthostatic hypotension test. The estimated jugular venous pressure was determined to be the distance between the oscillating top of the internal jugular venous pulsation and the sternal angle, using a head elevation angle of 30° to 45°. The orthostatic hypotension test was performed by measuring the BP of the patient in a prone position followed by the BP of the patient in a sitting position, measured within two to five minutes of sitting up. Fluid therapy was defined as adequate with an estimated jugular venous pressure >3 cm and/or a drop in systolic BP < 20 mmHg and/or a drop in diastolic BP < 10 mmHg.

Lung ultrasonography was performed for all patients with dengue shock, with the patient in a supine position. A cardiac transducer (1.5–3.5 MHz, GE Logiq S6, UK) was used for analyzing the anterior and lateral hemithoraxes, scanning along the parasternal, midclavicular, anterior axillary, and mid axillary lines, from the second to the fifth intercostal space on the right hemithorax and from the second to the fourth intercostal space on the left hemithorax. A total of 28 chest sites were scanned and the total number of B-lines was recorded.

### Fluid therapy

According to the WHO South East Asia Regional Organization (SEARO) guidelines, an inverted 'V' pattern of fluid therapy was recommended for patients with dengue, due to the dynamic fluid changes during the critical phase and recovery phase [1]. Patients without dengue shock received 0.9% or 0.45% sodium chloride with or without dextrose, at a rate adjusted based on their clinical condition, vital signs, urine output, and Hct level [1]. Patients with dengue shock initially received isotonic crystalloid at a rate of 300–500 mL over one hour or by bolus followed by an intravenous fluid rate based on their clinical condition, vital signs, urine output, and Hct level [1]. Further fluid therapy using 5% albumin was prescribed when additional fluid resuscitation was needed for patients with dengue shock or those without dengue shock who developed clinical fluid accumulation.

### Sample size calculation

We estimated the required sample size using recently published data [10]. As PP was used as one of the hemodynamic parameters for identifying circulatory shock among patients with dengue, we used the previous study results showing a mean change in PP of 10 mmHg during the defervescence period in our study. We assumed a standard deviation (SD) of 6 mmHg and the limits of the 95% CI were expected to be no more than 1 mmHg above or below the mean change in PP. The sample size was calculated using the Power and Sample Size Program, version 3.0, 2009 (Dupont WD, Creative Commons Attribution-NonCommercial-NoDerivs 3.0 United States License). This calculation found that we needed to study a minimum of 139 patients with dengue. We expected 10% of patients may refuse to participate or drop out before the study ends. Thus, a sample size of at least 155 patients with dengue was required for this study.

### Statistical analyses

Data were analyzed using SPSS for Windows 18.0 (IBM Corp., Chicago, IL). Numerical variables were tested for normality using Kolmogorov-Smirnov tests. Variables with non-normal distributions were summarized with medians and inter-quartile ranges (IQR) or 95% CI, as appropriate. The two independent groups were compared using Mann-Whitney *U* tests, and paired data were analyzed by the Wilcoxon Signed Ranks test. Categorical variables were expressed as frequencies and percentages and analyzed with chi-squared tests or Fisher’s exact tests, as appropriate. The Bland-Altman method was used for the intraobserver agreement of the measurements of IVCdmax and IVCdmin, using the method described above, by one well-trained investigator [35]. The same investigator repeated these measurements after an interval of no longer than 5 min. The 95% limits of agreement were calculated as the mean difference ± 1.96 SD of the difference. The intraclass correlation coefficient (ICC) for each parameter was calculated with a 95% CI. All tests of significance were two-sided with *P* <0.05 indicating statistical significance.

## Results

**Fig 1:**
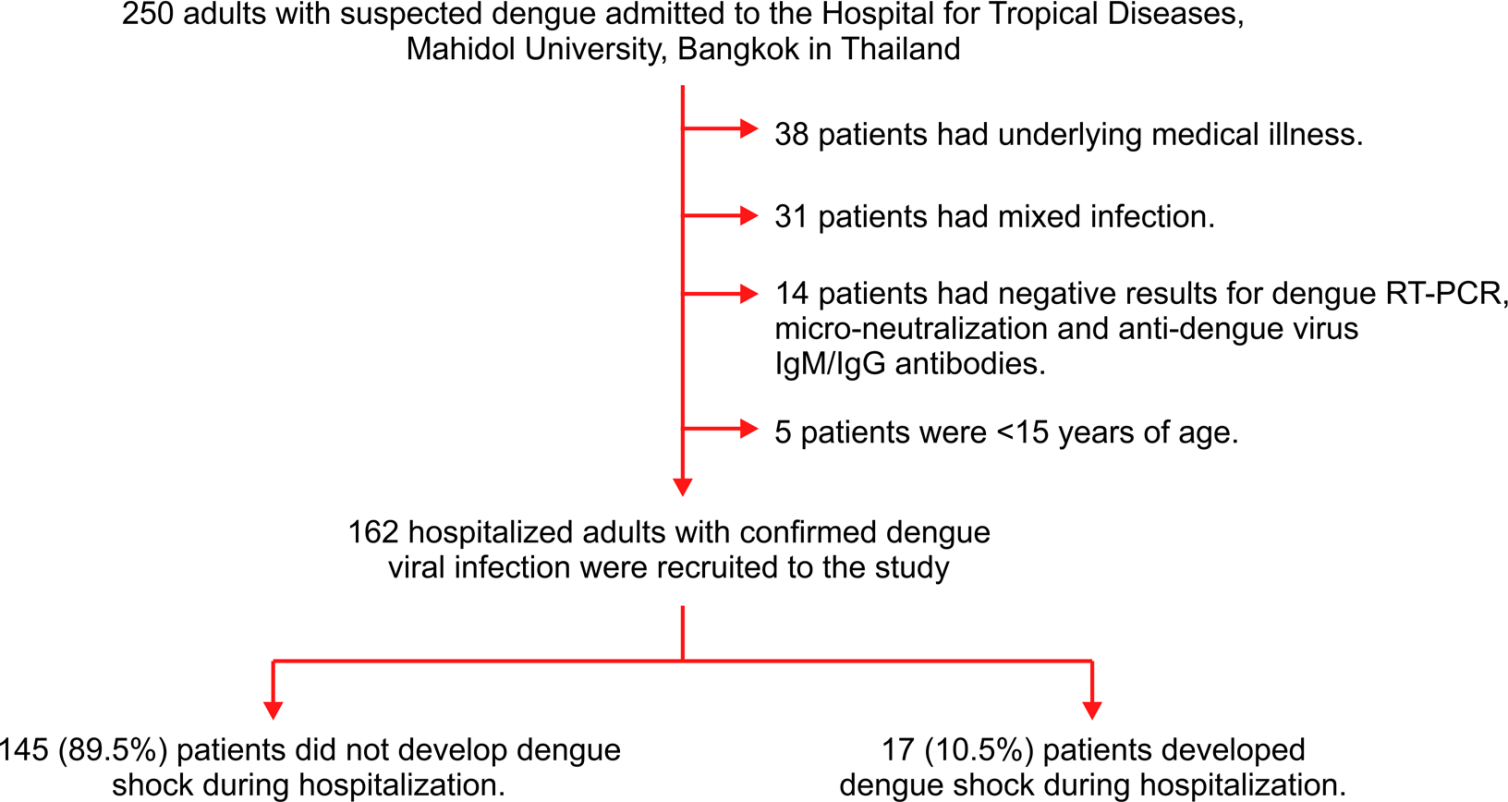
Flow diagram for the recruitment of study patients.

Of 250 patients with suspected dengue admitted to our hospital during the study period, 88 patients were excluded due to a history of underlying medical illness (N = 38), including hypertension (N = 11, 28.9%), diabetes mellitus (N = 8, 21.0%), lung diseases (N = 5, 13.2%), kidney diseases (N = 5, 13.2%), cardiovascular diseases (N = 4, 10.5%), liver diseases (N = 3, 7.9%), autoimmune diseases (N = 3, 7.9%) and HIV infection (N = 2, 5.3%); mixed infection (N = 31); negative results for dengue RT-PCR, micro-neutralization test, and anti-dengue virus IgM/IgG antibodies using ELISA (N = 14); and age <15 years (N = 5). Therefore, a total of 162 hospitalized adults with confirmed dengue viral infection were recruited to participate in the study (Fig 1). Of the 162 patients, all acute serum samples were assessed using dengue RT-PCR, and dengue serotypes were identified in 111 (68.5%) patients, including dengue serotypes 2 or 3 in 69 (62.2%) patients and dengue serotypes 1 or 4 in 32 (28.8%) patients. For the 51 patients with negative results for dengue RT-PCR, a micro-neutralization test was performed. Dengue serotypes could be identified in 50 (98.0%) patients, including dengue serotypes 1 or 4 in 33 (66.0%) and dengue serotypes 2 or 3 in 27 (54.0%); only one patient had undetermined dengue serotypes. The serological response to dengue viral infection was assessed using ELISA in 131 patients for whom paired serum samples were available, which identified 126 (77.8%) patients with secondary dengue infection and 5 (3.1%) patients with primary dengue infection. During the hospitalization period, 17 (10.5%) patients developed dengue shock and 145 (89.5%) did not.

### Comparison between patients with and without dengue shock

**Fig 2:**
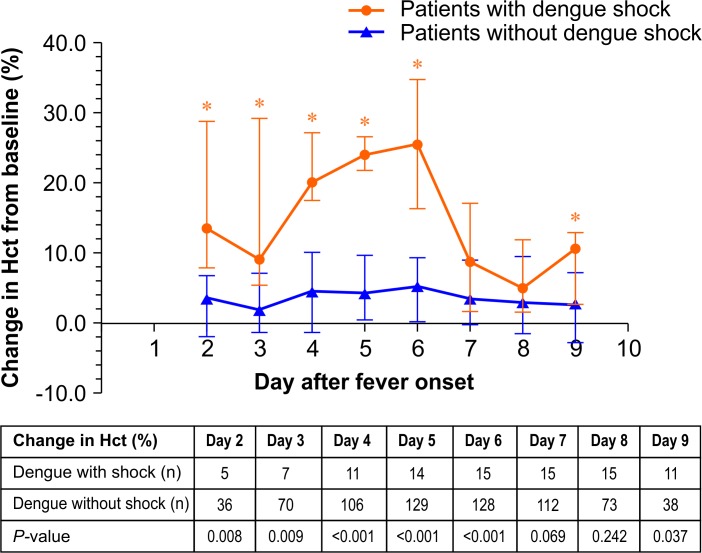
Change in hematocrit (Hct) from baseline (%) by day after fever onset among patients with and without dengue shock.

Baseline characteristics, clinical parameters, and laboratory parameters at admission are shown in Table 1. Patients with dengue shock were more likely to (1) be older (*P* = 0.022), (2) present with mucosal bleeding (*P* = 0.006), (3) have a liver span >15 cm (*P* = 0.017), (4) present with persistent vomiting (*P* = 0.034), and (5) have clinical fluid accumulation (*P* = 0.001). Patients with dengue shock had a significantly higher (1) change in Hct from baseline (*P* = 0.023), (2) AST level (*P* = 0.002), and (3) ALT level (*P* = 0.016), but had a significantly lower platelet count (*P* = 0.016) and serum albumin level (*P* <0.001). For the purposes of this study, the day of fever onset is considered day 1. Body temperature decreased to a normal level (<37.8°C) on day 6, with a median [IQR] body temperature of 37.2 [37.0–37.8] °C. Changes in Hct from baseline were significantly higher among patients with dengue shock than those without on days 2–6 and day 9 after fever onset (*P* <0.05) (Fig 2).

**Table 1:** Baseline characteristics, clinical parameters, laboratory parameters, and outcomes among 162 hospitalized adults with dengue, subdivided by patients with and without dengue shock.

Characteristics	All dengue cases	Dengue with shock	Dengue without shock	*P*-value
	(n = 162)	(n = 17)	(n = 145)	
*Baseline characteristics*				
Age (years)	24.5 (19.0–36.0)	34.0 (21.5–47.0)	23.0 (19.0–34.0)	0.022
Sex, male	87 (53.7)	9 (52.9)	78 (53.8)	1.000
*Clinical parameters*				
Fever (days)	4.0 (3.0–5.0)	5.0 (3.0–5.5)	4.0 (3.0–5.0)	0.284
Skin bleeding	92 (56.8)	13 (76.5)	79 (54.5)	0.141
Mucosal bleeding	78 (48.1)	14 (82.4)	64 (44.1)	0.006
Liver span >15 cm	66 (40.7)	12 (70.6)	54 (37.2)	0.017
Persistent vomiting	26 (16.0)	6 (35.3)	20 (13.8)	0.034
Clinical fluid accumulation	25 (15.4)	8 (47.1)	17 (11.7)	0.001
Temperature (°C)	38.4 (37.7–39.1)	38.5 (37.4–39.2)	38.4 (37.8–39.1)	0.915
Mean arterial pressure (mmHg)	84 (77–92)	83 (69–88)	85 (78–92)	0.116
Heart rate (beats/min)	79 (67–88)	84 (68–92)	78 (66–88)	0.202
*Confirmation tests for dengue*				
Dengue serotypes, positive	161 (99.4)	17 (100)	144 (99.3)	1.000
Serotypes 1 or 4	65 (40.1)	7 (41.2)	58 (40.0)	
Serotypes 2 or 3	96 (59.2)	10 (58.8)	86 (59.3)	
Dengue ELISA^a^, positive	131 (100)	15 (100)	116 (100)	0.461
Primary dengue infection	5 (3.1)	1 (5.9)	4 (2.8)	
Secondary dengue infection	126 (77.8)	14 (82.3)	112 (77.2)	
*Laboratory parameters*				
Hemoglobin, g/dL	13.9 (12.9–15.0)	13.8 (11.8–17.6)	13.9 (12.9–15.0)	0.855
Hematocrit, %	41.6 (38.2–44.4)	42.0 (35.4–50.6)	41.6 (38.6–44.4)	0.831
Percent change in hematocrit^b^, %	3.9 (-1.7–10.2)	9.0 (0.5–21.3)	4.0 (-2.4–9.8)	0.023
WBC, ×10^3^/μL	3.4 (2.4–5.0)	4.0 (2.2–6.0)	3.3 (2.4–4.9)	0.422
Platelet count, ×10^3^/μL	84.0 (47.8–131.5)	56.0 (31.5–82.0)	85.0 (53.0–139.5)	0.016
Creatinine, mg/dL	0.8 (0.6–1.0)	0.8 (0.6–1.0)	0.8 (0.7–1.0)	0.883
Albumin, g/dL	4.3 (3.9–4.5)	3.9 (3.2–4.2)	4.3 (4.0–4.5)	<0.001
AST, IU/L	82 (37–182)	219 (82–391)	77 (36–157)	0.002
ALT, IU/L	54 (22–118)	84 (59–240)	47 (20–107)	0.016
*Management*				
Isotonic crystalloid	160 (98.8)	17 (100)	143 (98.6)	1.000
5% albumin	12 (7.4)	9 (52.9)	3 (2.1)	<0.001
Blood components	8 (4.9)	4 (23.5)	4 (2.8)	0.005
*Outcomes*				
Abnormal ECG	65 (40.1)	8 (47.1)	57 (39.3)	0.722
Elevated NT-proBNP ≥500 pg/mL	23 (14.2)	6 (35.3)	17 (11.7)	0.018
Elevated Troponin T	2 (1.2)	2 (11.8)	0	0.010
New onset or increased pleural effusion^c^	28 (19.0)	9 (52.9)	19 (14.6)	0.001
Acute kidney injury^d^	16 (9.9)	6 (35.5)	10 (6.9)	0.002
Duration of hospitalization (days)	3.7 (2.7–4.9)	4.7 (2.8–8.3)	3.5 (2.7–4.8)	0.041
Death	2 (1.2)	2 (11.8)	0	0.010

Data are presented as the N (%) or median (IQR), as appropriate.

^a^Dengue ELISA was performed in 131 patients, including 15 with and 116 without dengue shock.

^b^Percent change in hematocrit from baseline, from a total of 160 patients, including 15 patients with and 145 patients without dengue shock.

^c^The occurrence of new onset or increased pleural effusion was determined by chest radiograph, with a total number of 147 patients.

^d^Acute kidney injury was defined as (1) an increase in serum creatinine by ≥0.3 mg/dL within 48 hours or (2) an increase in serum creatinine ≥1.5 times baseline.

ALT = alanine aminotransferase; AST = aspartate aminotransferase; ECG = electrocardiography; ELISA = enzyme-linked immunosorbent assays; IQR = interquartile range; NT-proBNP = N-terminal pro-brain natriuretic peptide; WBC = white blood cell count.

Regarding the management and outcomes of the study patients (Table 1), a significantly higher proportion of patients with dengue shock received 5% albumin (*P* <0.001) and blood components (*P* = 0.005) compared to patients without dengue shock. In addition, patients with dengue shock had: (1) an elevated rate of NT-proBNP ≥500 pg/mL (*P* = 0.018), (2) elevated troponin T levels (*P* = 0.010), (3) a higher number of cases with new onset or increased pleural effusion, assessed by chest radiography (*P* = 0.001), (4) an increased likelihood of developing acute kidney injury (AKI), defined as an increase in serum creatinine by ≥0.3 mg/dL within 48 hours or an increase in serum creatinine to ≥1.5 times baseline (*P* = 0.002), (5) a longer hospitalization duration (*P* = 0.041), and (6) higher in-hospital mortality (*P* = 0.010).

### Hemodynamic parameters

**Fig 3:**
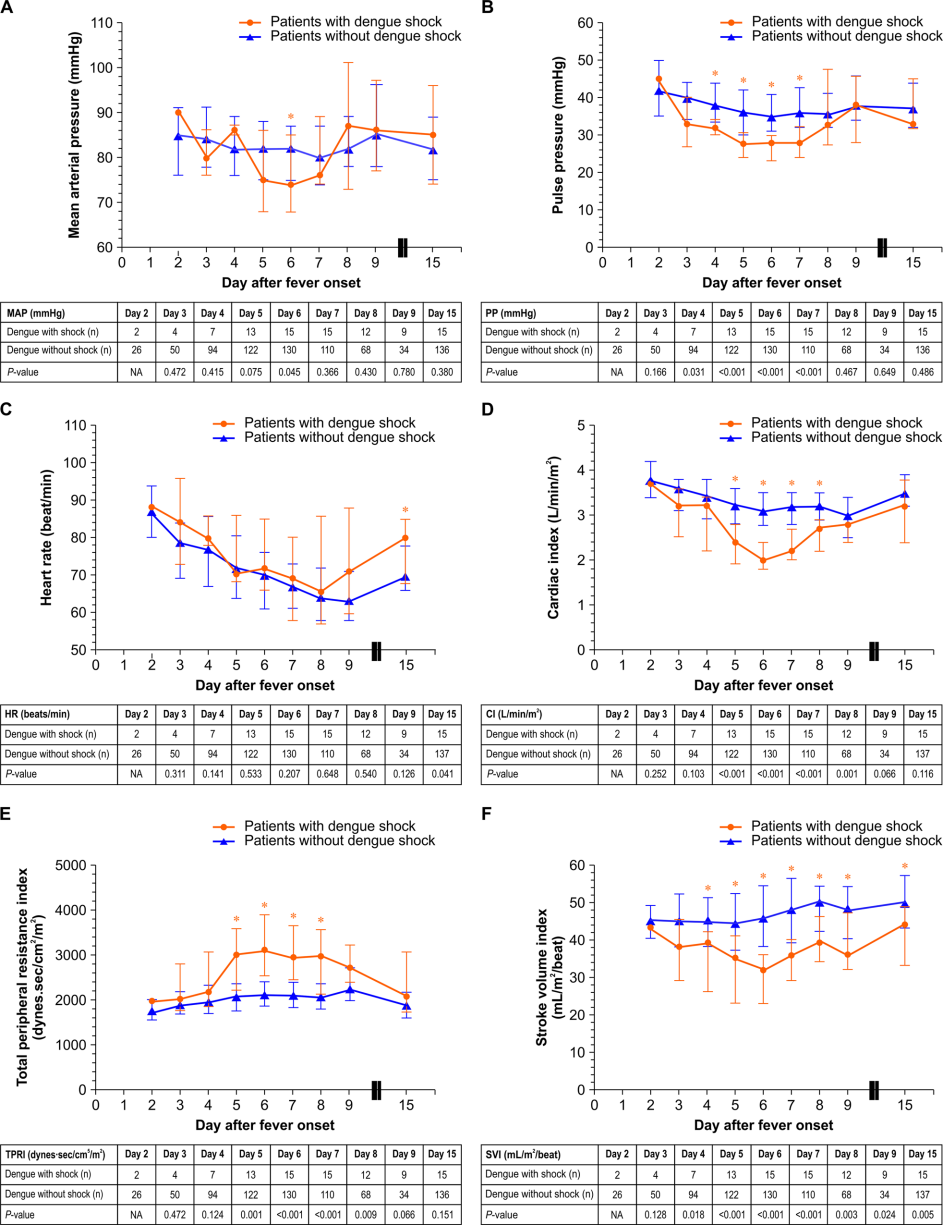
Hemodynamic parameters by day after fever onset among patients with and without dengue shock. (A) MAP (mmHg) after fever onset among patients with and without dengue shock. (B) PP (mmHg) after fever onset among patients with and without dengue shock. (C) HR (beats/min) after fever onset among patients with and without dengue shock. (D) CI (L/min/m^2^) after fever onset among patients with and without dengue shock. (E) TPRI (dynes∙sec/cm^5^/m^2^) after fever onset among patients with and without dengue shock. (F) SVI (mL/m^2^/beat) after fever onset among patients with and without dengue shock. CI = cardiac index; HR = heart rate; MAP = mean arterial pressure; NA = not applicable; PP = pulse pressure; SVI = stroke volume index; TPRI = total peripheral resistance index.

Regarding the primary hemodynamic parameters, MAP was similar in both groups on all days except day 6, when a significantly lower MAP was observed among patients with dengue shock (Fig 3A). PP was significantly lower among patients with dengue shock between days 4 and 7 (*P* <0.05) (Fig 3B). HR was similar in both groups except on day 15, when a significantly higher HR was observed among patients with dengue shock (*P =* 0.041) (Fig 3C). Examining the advanced hemodynamic parameters revealed that patients with dengue shock had a significantly lower CI (Fig 3D), but higher TPRI (Fig 3E), between days 5 and 8 (*P* <0.05). In addition, SVI was significantly lower among patients with dengue shock between days 4 and 15 (*P* <0.05) (Fig 3F).

### Daily central venous pressure assessment

**Fig 4:**
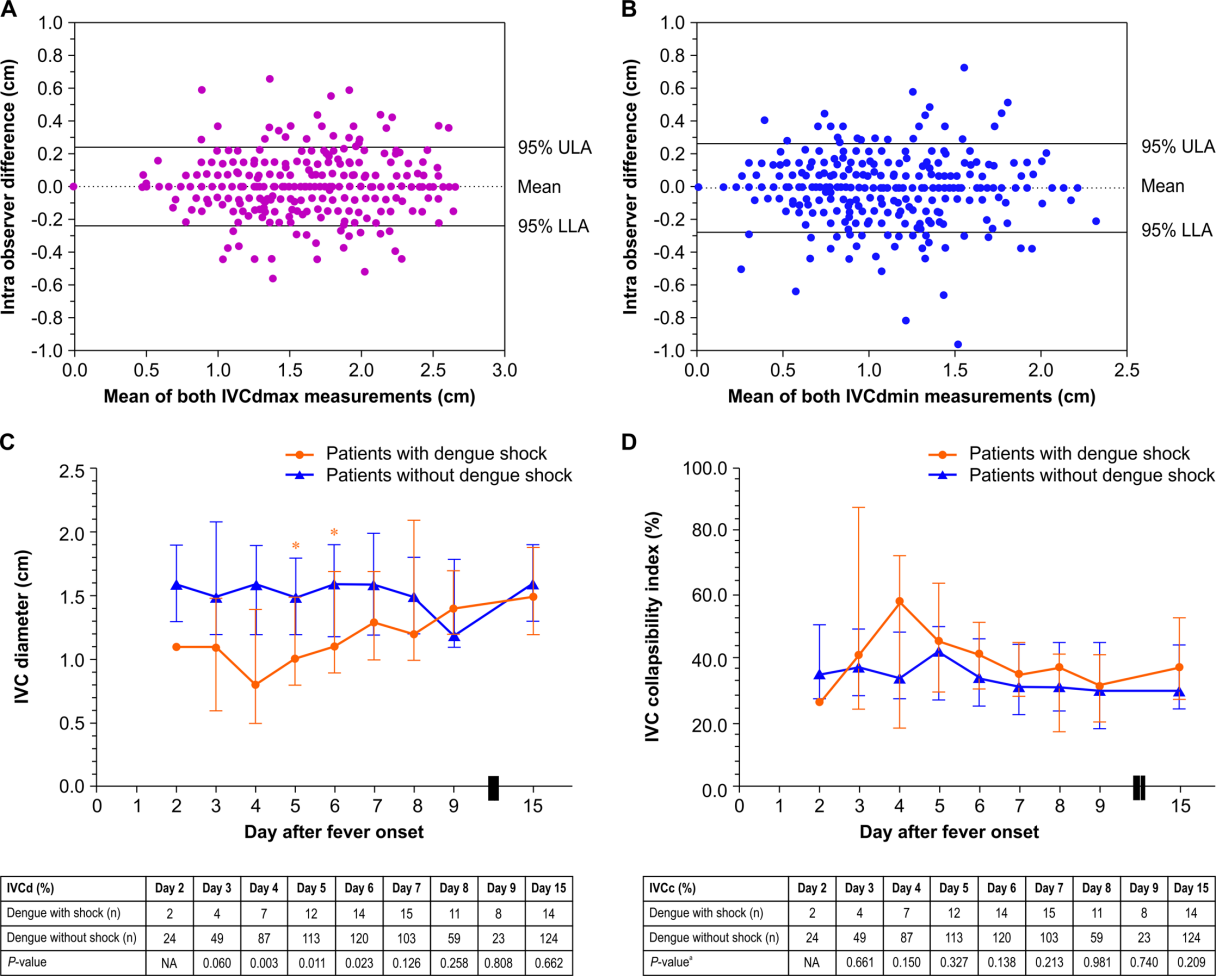
Inferior vena cava parameters by day after fever onset among patients with and without dengue shock. (A) A Bland-Altman plot of data from the intraobserver reliability study. The mean IVCdmax (cm) of each patient was plotted against the difference in IVCd (cm) between two measurements (811 measurements each) obtained by the same observer. (B) A Bland-Altman plot of data from the intraobserver reliability study. The mean IVCdmin (cm) of each patient was plotted against the difference in IVCd (cm) between two measurements (811 measurements each) obtained by the same observer. (C) IVCd (cm) after fever onset among patients with and without dengue shock (D) IVCc (%) after fever onset among patients with and without dengue shock. IVCc = Inferior vena cava collapsibility index; IVCd = inferior vena cava diameter; IVCdmax = maximum inferior vena cava diameter; IVCdmin = minimum inferior vena cava diameter; LLA = lower limit of agreement; NA = not applicable; ULA = upper limit of agreement.

A Bland-Altman plot showed narrow limits of agreement (Fig 4A and 4B); the mean difference between the first and second measurements was 0.008 cm (95% CI -0.229–0.244 cm) for IVCdmax and -0.004 cm (-0.274–0.266 cm) for IVCdmin. To further assess the agreement, the ICC was calculated and found to be 0.966 (95% CI 0.961–0.970) for IVCdmax and 0.945 (0.937–0.952) for IVCdmin, indicating excellent agreement between the first and second measurements. Evaluation of IVCd and IVCc were performed to estimate CVP. Significantly lower IVCd values were observed among patients with dengue shock between days 4 and 6 (*P* <0.05) (Fig 4C), but IVCc was similar in both groups (Fig 4D). Based on the categorized IVC parameters that correspond to a CVP ≤5 mmHg, the proportion of dengue shock patients who had IVCd <1.5 cm and IVCc >50% was significantly higher on day 4 (4/7 [57.1%] patients vs. 10/87 [11.5%] patients, *P* = 0.009) and day 5 (5/12 [41.7%] patients vs. 18/113 [15.9%] patients, *P* = 0.044).

### Modified approach protocol for assessing types of circulatory failure

**Fig 5:**
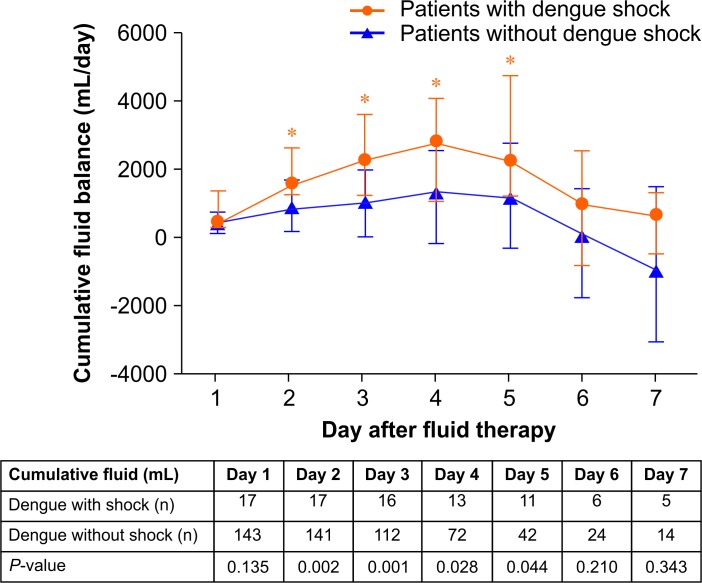
Daily cumulative fluid balance (mL) by day after fluid therapy among patients with and without dengue shock.

Dengue shock occurred in 17 patients, at a median (IQR) time of 6.0 (5.0–6.5) days after fever onset. All 17 patients had plasma leakage and 8 (47.0%) had clinical fluid accumulation, manifested as pleural effusion (5 [62.5%] patients) and serum albumin <3.5 g/dL (5 [62.5%] patients). All patients with dengue shock had a systolic BP of <90 mmHg with evidence of tissue hypoperfusion, including a urine output <0.5 mL/kg/h (9 patients, 52.9%), AST or ALT >1,000 IU/L (6 patients, 35.3%), impaired consciousness (4 patients, 23.5%), and cold or clammy skin (2 patients, 11.8%). In addition, 4 (23.5%) patients presented with a rapid, weak pulse and PP <20 mmHg. Using a modified approach protocol for determining the type of circulatory shock, we detected hypovolemic shock in 9 (52.9%) patients and cardiogenic shock in 8 (47.1%) patients. Patients with cardiogenic shock had significantly higher NT-proBNP (median [IQR]: 970.5 [678.0–7036.8] pg/mL vs. 141.0 [107.5–218.0] pg/mL, *P* <0.001) and total number of B-lines (36 [21–81] scores vs. 3 [0–8] scores, *P* = 0.008) than those with hypovolemic shock. After resuscitation, 3 (17.6%) patients had recurrent dengue shock caused by cardiogenic shock. The daily cumulative fluid balance was significantly higher among patients with dengue shock between days 2 and 5 (*P* <0.05) (Fig 5).

## Discussion

Hemodynamic monitoring is a functional tool used for assessing the pathophysiological process of a disease, and proper monitoring can alert health care teams to an impending cardiovascular crisis before the development of organ injury [12]. It is also used to facilitate diagnosis, which enables more effective management, and to monitor response to therapy [12, 13]. The appropriate hemodynamic monitoring modality depends on the availability of techniques in the treating institution, the accuracy of the devices, and the patient’s condition and contraindications [14]. Patients with dengue are at risk for bleeding, thus non-invasive hemodynamic monitoring should be considered for serial assessments among patients with dengue shock.

We conducted a prospective observational study among hospitalized adults with dengue using a non-invasive CO monitor, based on the bioreactance technique, and 2D bedside ultrasonography. Previous studies have shown that the bioreactance technique provides high accuracy for assessing advanced hemodynamic parameters compared to standard methods, particularly among relatively stable patients [24–28]. Recently, bedside ultrasonography protocols have been established and are widely used for identifying types of circulatory shock, as well as for guiding for fluid therapy among patients with circulatory shock. This technique utilizes IVC parameters and lung ultrasound to estimate the cardiac preload and extravascular lung fluid, respectively [16, 17, 36]. However, these non-invasive bedside techniques have not been extensively investigated among patients with dengue. Therefore, a prospective observational study was conducted among hospitalized adults with dengue to determine the changes in hemodynamic and IVC parameters and to identify types of circulatory shock among patients with dengue using non-invasive bedside techniques.

In this study, 17 (10.5%) patients had dengue shock. On admission, patients with dengue shock were more likely to: (1) be older, (2) have mucosal bleeding, (3) have an enlarged liver >15 cm, (4) have persistent vomiting, and (5) have clinical fluid accumulation. Laboratory findings among patients with dengue shock showed (1) a higher percent change in Hct above the reference range, (2) lower platelet counts, (3) lower serum albumin levels, and (4) elevated liver enzymes. Similarly, a number of reports showed that age >37 years, presence of bleeding, low total protein level, elevated liver enzymes ˃3-fold above the reference range, and >2% change in Hct above the reference range were independently associated with severe dengue [8, 37–40].

Regarding the primary hemodynamic parameters, patients with dengue shock had a significantly lower MAP at day 6, which was the day of defervescence. Patients with shock also had a significantly lower PP between days 4 and 7 (28–32 mmHg). A previous study from Vietnam showed a significantly lower PP among adults with severe dengue compared to those with non-severe dengue (30 mmHg vs. 40 mmHg, *P* = 0.011) [10]. As arterial PP is directly related to the ventricular SV and inversely related to the vascular compliance, a lower PP among patients with dengue shock represents a decrease in SV [41]. Compared to patients with septic shock, patients with dengue shock had significantly lower PP and HR in a previous study from India [42]. At body temperatures of 39.0°C and 39.8°C, our patients with dengue shock had relatively slower HRs, of 80 and 88 beats/min, respectively. Similarly, a previous report from Singapore demonstrated relative bradycardia among patients with dengue [43].

Regarding advanced hemodynamic parameters, patients with dengue shock had significantly decreased SVI between days 4 and 15 and a significantly decreased CI between days 5 and 8. Similar to a previous study from Vietnam, patients with severe dengue had significantly lower SV and CO at admission (approximately day 5) due to the combination of systolic and diastolic dysfunction [10]. The group of patients with dengue shock in our study had a significantly higher proportion of patients with IVCd <1.5 cm and IVCc >50% between days 4 and 5. Previous studies have shown that IVCd <1.5 cm and IVCc >50% are correlated with a CVP ≤5 mmHg [31, 32]. It is likely that patients with dengue shock had intravascular hypovolemia due to ongoing plasma leakage between days 4 and 5. On the day of defervescence, approximately 6 days after fever onset, patients with dengue shock had a minimum CI and SVI, but a maximum TPRI. However, the proportions of patients with IVCd <1.5 cm and IVCc >50% in both groups were similar. Thus, it is likely that patients with dengue shock developed myocardial dysfunction on the day of defervescence that persisted until day 15. The median duration before the occurrence of dengue shock was 6 days after fever onset. The causes of dengue shock in our study were hypovolemic shock and cardiogenic shock, each occurring in approximately half of the patients with dengue shock. A study from India found that 62.5% of dengue patients with circulatory shock had a left ventricular ejection fraction <40% [8]. Patients with cardiogenic shock in our study had elevated NT-proBNP, with a median level of 970.5 pg/mL, which is above the level associated with acute cardiogenic pulmonary edema among patients aged ≤75 years [44]. A previous study showed that the level of NT-proBNP was significantly correlated with the number of B-lines (r = 0.69, *P* <0.001), and the receiver operating characteristic analysis showed an area under the curve of 0.978 for NT-proBNP and 0.893 for the number of B-lines for identifying patients with acute cardiogenic pulmonary edema [45]. According to the Bedside Lung Ultrasound in Emergency (BLUE) and Fluid Administration Limited by Lung Sonography (FALLS) protocols, B-lines could be used to differentiate cardiogenic shock from other types of shock [17]. In our study, patients with cardiogenic shock had a median of 36 B-lines, which is in the range of scores for a severe degree of pulmonary edema in a previous report (>30 scores) [46]. It is possible that IVC parameters and lung ultrasound according to the Point-Of-Care Ultrasound (POCUS) could be used as guides to manage fluid therapy among patients with dengue, particularly patients with dengue shock, to avoid insufficient or excessive fluid resuscitation [36].

Intravascular fluid administration is the key intervention for dengue shock, serving to counteract plasma leakage from capillaries during the critical phase [3]. An inverted 'V' pattern of fluid therapy is recommended by the WHO SEARO guidelines for patients with dengue, due to dynamic fluid changes during the critical and recovery phases [1]. Inappropriate fluid therapy increases the risk of fluid overload and congestive heart failure among patients with severe plasma leakage [1, 3]. However, patients who receive inadequate intravascular fluid administration might progress to shock during the critical phase [1, 3]. Both fluid overload and inadequate intravascular volume could lead to worsening of tissue oxygenation, resulting in organ failure among patients with dengue [47]. In our study, an inverted 'V' pattern was used for guiding fluid therapy, according to the WHO SEARO guidelines [1]. However, patients with dengue shock had a significantly higher cumulative fluid balance between days 2 and 5 after fluid therapy. Previous reports showed that in children with dengue shock, receiving colloid (i.e., dextran 70, gelafundin, or 6 percent hydroxyethyl starch) restored cardiovascular stability more rapidly than isotonic crystalloid [48–50]. However, a report showed that patients with shock who received artificial hyperoncotic colloids and hyperoncotic albumin as fluid therapy were at risk for AKI [51]. In our study, isotonic crystalloid was used for initial fluid resuscitation and 5% albumin was used when further fluid resuscitation was needed. After resuscitation, approximately 17.6% of patients with dengue shock experienced recurrent cardiogenic shock; this rate is lower than that reported in children (28.0%) [52].

Regarding the outcomes of dengue patients in our study, patients with dengue shock were more likely to: (1) have elevated NT-proBNP, (2) have an elevated troponin T level, (3) develop new onset or increased pleural effusion, (4) have AKI, (5) have a longer hospitalization duration, and (6) have higher in-hospital mortality. These results are similar to previous studies [8, 10, 11]. Among patients with dengue shock, new onset pleural effusion or an increased pleural fluid level was observed in 52.9%, which is similar to previous studies [10, 11]. AKI was observed in 35.5% of patients with dengue shock, 50% of whom received renal replacement therapy. A previous report showed that 71.4% of adults with fatal dengue due to dengue shock and organ failure had AKI, defined as a serum creatinine level >2 fold larger than the upper normal limit, though the majority of patients had comorbid diseases [53]. However, in-hospital mortality in our study (1.2%) was lower than values presented in a previous report (2.5–5.0%) of patients with dengue [54].

Our study has some limitations. (1) This study was conducted in a single center, which may limit its generalizability, though the study center is the referral center for tropical diseases in Thailand. (2) The patients in this study were not admitted on a uniform day after fever onset, and data prior to hospitalization are limited. However, all patients were admitted during the febrile phase of dengue. (3) Echocardiographic data were not collected in this study, due to the limited availability of an expert echocardiographer and patient discomfort. At present, non-invasive CO monitoring and bedside ultrasonography are used to provide better care for critically ill patients by enabling timely recognition of worsening symptoms and improved diagnostic ability, promoting the proper management of cases. However, further study is needed to compare the outcomes of patients with dengue shock using standard treatment versus a pathophysiological approach with a bedside ultrasonography protocol for resuscitation in patients with dengue shock.

In conclusion, intravascular hypovolemia occurred before defervescence and myocardial dysfunction occurred on the day of defervescence and persisted until at least 2 weeks after fever onset. Cardiogenic and hypovolemic shock were the causes of shock in dengue. A dynamic approach that involves monitoring the pathophysiological process and preload assessment in the clinical phase of dengue could help physicians provide proper treatment, particularly fluid therapy, which might result in decreased complications due to dengue shock in the future.
